# 

**Published:** 1894-10-06

**Authors:** 


### CONTENTS.

**The Opening of the Medical Schools** - - - - - 1

**Passive Motion** - - - - - - - - - - - - - 2

**On Modern Progress in Ophthalmic Medicine and Surgery.** By Robert Brudenell Carter, F.R.C.S.—Laminar Cataract (*continued*) - - - - - - - - - 3

**Modern Medico-Psychology and Psychiatry.**—By T. S. Clouston, M.D.—XII. Mental States of Alternation, Rhythm, and Periodicity - - - - - - - - - - - - 4

**Annotations.**—The Illness of the Tzar.—The Teaching of Hygiene in Schools.—Ladies at Golf - - - - - - - 5

**Notes and News** - - - - - - - - - - - - 6

**Medical Progress and Hospital Clinics:**

Notes on the Methods of Performing Paracentesis of the various Cavities - - - - - - - - - - 7

Progress in Medicine.—Anæsthetics - - - - - - 9

Progress in Surgery.—General Surgery - - - - - 11

New Appliances and Things Medical - - - - - 12

**The Institutional Workshop:**

Hospital Construction - - - - - - - - - 13

The Story of the Insane from Year to Year - - - 14

Practical Departments, 15; Hospital Meetings - - - 16

**Scraps and Gleanings** - - - - - - - - - - 16

### EXTRA SUPPLEMENT.—THE NURSING MIRROR.

**News from the Nursing World.**—"Thank God there are Nurses!"—Dr. James Anderson—Maternity Work.—A Valuable Citizen.—A Nurse's Diagnosis.—Nursing at Chippenham.—Pleasant Instruction.—Soldiers' Wives.—An Independent Institution.—Queen's Nurse at Forfar.—Diverting the Patients.—The McLean Hospital—Short Items - - - - - - - - i

**Our Work** - - - - - - - - - - - - iii

**"The Hospital" Convalescent Fund** - - - - - - iii

**Scattered Nurses—District Nursing** - - - - - iv

**Nursing in Metropolitan Fever Hospitals** - - - - v

**Army Sisters—Mental Nurses** - - - - - - - - v

**Nursing in Workhouses—Where to Go** - - - - - v

**Dress and Uniforms** - - - - - - - - - - vi

**A Novel Competition** - - - - - - - - - - viii

**A Visit to Edmonton Workhouse** - - - - - - - ix

**Massage** - - - - - - - - - - - - - ix

**Trained Nurses as Stewardesses—Male Nurses** - - - x

**Appointments—Wants and Workers** - - - - - - x

**Wigmore Co-operative Nursing Institution** - - - - x

**Novelties for Nurses—Massage** - - - - - - - x

